# Unbound and Periprostatic Adipose Tissue Cefazolin Pharmacokinetics in Robotic-Assisted Radical Prostatectomy

**DOI:** 10.3390/antibiotics15020181

**Published:** 2026-02-06

**Authors:** Toshiaki Komatsu, Yuki Takahashi, Yoko Takayama, Yuto Akamada, Masaomi Ikeda, Hideyasu Tsumura, Daisuke Ishii, Kazumasa Matsumoto, Masatsugu Iwamura, Hirotsugu Okamoto, Hideaki Hanaki, Katsuya Otori

**Affiliations:** 1 Department of Pharmacy, Kitasato University Hospital, Sagamihara 252-0375, Kanagawa, Japan; akamad.y@kitasato-u.ac.jp (Y.A.); kotori@kitasato-u.ac.jp (K.O.); 2 Pharmacy Practice and Science I, Research and Education Center for Clinical Pharmacy, Kitasato University School of Pharmacy, Sagamihara 252-0375, Kanagawa, Japan; pp20152@st.kitasato-u.ac.jp; 3 Department of Infection Control and Infectious Diseases, Research and Development Center for New Medical Frontiers, Kitasato University School of Medicine, Sagamihara 252-0374, Kanagawa, Japan; yoko@med.kitasato-u.ac.jp; 4 Department of Urology, Kitasato University School of Medicine, Sagamihara 252-0374, Kanagawa, Japan; ikeda.masaomi@grape.plala.or.jp (M.I.); tsumura@med.kitasato-u.ac.jp (H.T.); daisukei@med.kitasato-u.ac.jp (D.I.); kazumasa@cd5.so-net.ne.jp (K.M.); miwamura@med.kitasato-u.ac.jp (M.I.); 5 Department of Urology, Tomei Atsugi Hospital, Atsugi 243-0035, Kanagawa, Japan; 6 Departments of Pharmacology and Anesthesiology, Kitasato University School of Medicine, Sagamihara 252-0374, Kanagawa, Japan; okasuke@med.kitasato-u.ac.jp; 7 Ōmura Satoshi Memorial Institute, Kitasato University, Minato-ku, Tokyo 108-8641, Japan; hanaki@insti.kitasato-u.ac.jp

**Keywords:** cefazolin, population pharmacokinetics, periprostatic adipose tissue, robotic-assisted radical prostatectomy, surgical site infection

## Abstract

**Background/Objectives:** This study aimed to describe the population pharmacokinetics of cefazolin (CFZ) using unbound serum and periprostatic adipose tissue concentrations and to optimize dosing regimens for patients undergoing robotic-assisted radical prostatectomy (RARP). **Methods:** We investigated the population pharmacokinetics of CFZ using 295 unbound serum and 67 periprostatic adipose tissue samples from 67 individuals. CFZ concentrations were determined in all samples. A nonlinear mixed-effects model was developed. The pharmacodynamic target was defined as maintaining unbound trough and periprostatic adipose tissue concentrations exceeding the minimum inhibitory concentration (MIC) against methicillin-susceptible *Staphylococcus aureus* (MSSA) for over 90% of the dosing interval (MIC_90_; 0.5 mg/L). **Results:** Systemic clearance of unbound CFZ was significantly associated with creatinine clearance (CLcr). In patients with normal renal function, simulations showed that a 1 g CFZ infusion over 15 min maintained unbound concentrations exceeding the MSSA MIC_90_ for >90% of the 3 h interval after the initial dose. Notably, in patients with mild renal impairment (CLcr ≤ 80 mL/min), a 5 h dosing interval also achieved a >90% probability of maintaining the target CFZ concentration. **Conclusions:** The simulations demonstrated that the probability of target attainment of >90% was maintained for up to 5 h in patients with mild renal impairment (CLcr ≤ 80 mL/min). These findings provide a pharmacokinetic rationale suggesting that the standard additional dose may not be necessary for this subgroup; however, future clinical studies are needed to validate safety and efficacy.

## 1. Introduction

Prostate cancer is the second most commonly diagnosed cancer among male individuals and the fifth leading cause of cancer-related deaths in men, according to global cancer statistics for 2022 [1]. Robotic-assisted radical prostatectomy (RARP) is currently the most prominent treatment option for men with localized prostate cancer, provided they have a life expectancy of at least 10 years and are deemed suitable candidates for curative intervention [2]. Although RARP is a minimally invasive procedure, surgical site infections (SSIs) remain a major concern, potentially leading to prolonged hospitalization, increased medical costs, and high patient morbidity [3,4]. As RARP is classified as a clean-contaminated surgery, effective antibiotic prophylaxis is critical for preventing these infectious complications [5].

Clinical guidelines primarily recommend first-generation cephalosporins, particularly cefazolin (CFZ) [3,4], which is routinely used for perioperative prophylaxis because it demonstrates strong antibacterial activity against methicillin-susceptible *Staphylococcus aureus* (MSSA) and other Gram-positive cocci [6,7]. To ensure effective prophylaxis, antimicrobial agents must maintain adequate concentrations not only in the serum but also specifically at the surgical site—the target tissue where infection is most likely to originate [8]. For RARP, the periprostatic adipose tissue represents a critical site of potential bacterial contamination. However, CFZ exhibits high and saturable protein binding (approximately 80–90%) [9,10]. As only the unbound fraction of the drug is pharmacologically active and capable of penetrating tissues, relying solely on total serum concentrations may misestimate the actual antimicrobial activity at the target site [11]. Therefore, measuring both unbound serum and specific tissue concentrations is essential for accurately assessing target attainment.

Despite the widespread use of CFZ, there are substantial knowledge gaps regarding its pharmacokinetics in the specific context of RARP. Current dosing recommendations (e.g., 3 h redosing intervals) are largely derived from studies on general surgical populations or pharmacokinetic data on other tissue types, such as the skin and bone [12,13]. Currently, there are no population pharmacokinetic models describing CFZ distribution specifically into periprostatic adipose tissue. Furthermore, the patient population undergoing RARP typically consists of older adult men who often present with age-related physiological changes, including diminished renal function, which can substantially alter CFZ clearance (CL) [14]. It remains unclear whether the standard “one-size-fits-all” redosing strategy is optimal for this specific demographic and tissue site. Therefore, we aimed to describe the population pharmacokinetics of CFZ using unbound serum and periprostatic adipose tissue concentrations to optimize dosing regimens specifically for patients undergoing RARP.

## 2. Results

**Figure 1:**
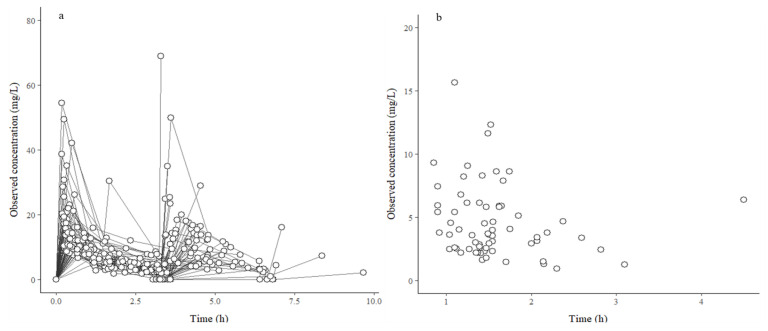
Observed cefazolin concentrations. Individual unbound serum (**a**) and periprostatic adipose tissue (**b**) cefazolin concentrations at various time points after dosing.

This study involved 67 male patients, whose baseline characteristics are presented in Table 1. In total, eight patients received 2 g CFZ. Unbound serum (*n* = 295) and periprostatic adipose tissue (*n* = 67) samples were analyzed, and CFZ concentrations at different time points are shown in Figure 1.

Among the structural models tested, the two-compartment model yielded a significantly lower Akaike information criterion (AIC) value than the one-compartment model, providing a better fit to the data. Regarding the residual error model, the proportional error model showed the lowest AIC value and was therefore identified as the optimal model. Inter-individual variability (IIV) for unbound intercompartmental clearance (Q) was initially estimated; however, the variance estimate was negligible and approached zero, leading to model instability. Consequently, the IIV for Q was fixed to zero in the final model. The final population parameter estimates are listed in Table 2. The covariates retained in the model were unbound CFZ clearance (CL) and creatinine clearance (CLcr) normalized to 69 mL/min. The final model was defined as follows:

CL (L/h) = 25.8 × (CLcr/69)^0.735^,

where the unbound volume of distribution of the central compartment (V_c_) was 44.3 L, Q (L/h) was 44.0 L/h, the unbound volume of distribution of the peripheral compartment (V_p_) was 52.2 L, and F_PA_ was 0.638.

The coefficients of variation for interindividual variability (ω2) for CL, V_c_, V_p_, and F_PA_, and residual variability (σ^2^) for unbound concentrations were 28.3, 52.9, 46.3, 45, and 21.6, respectively.

**Figure 2:**
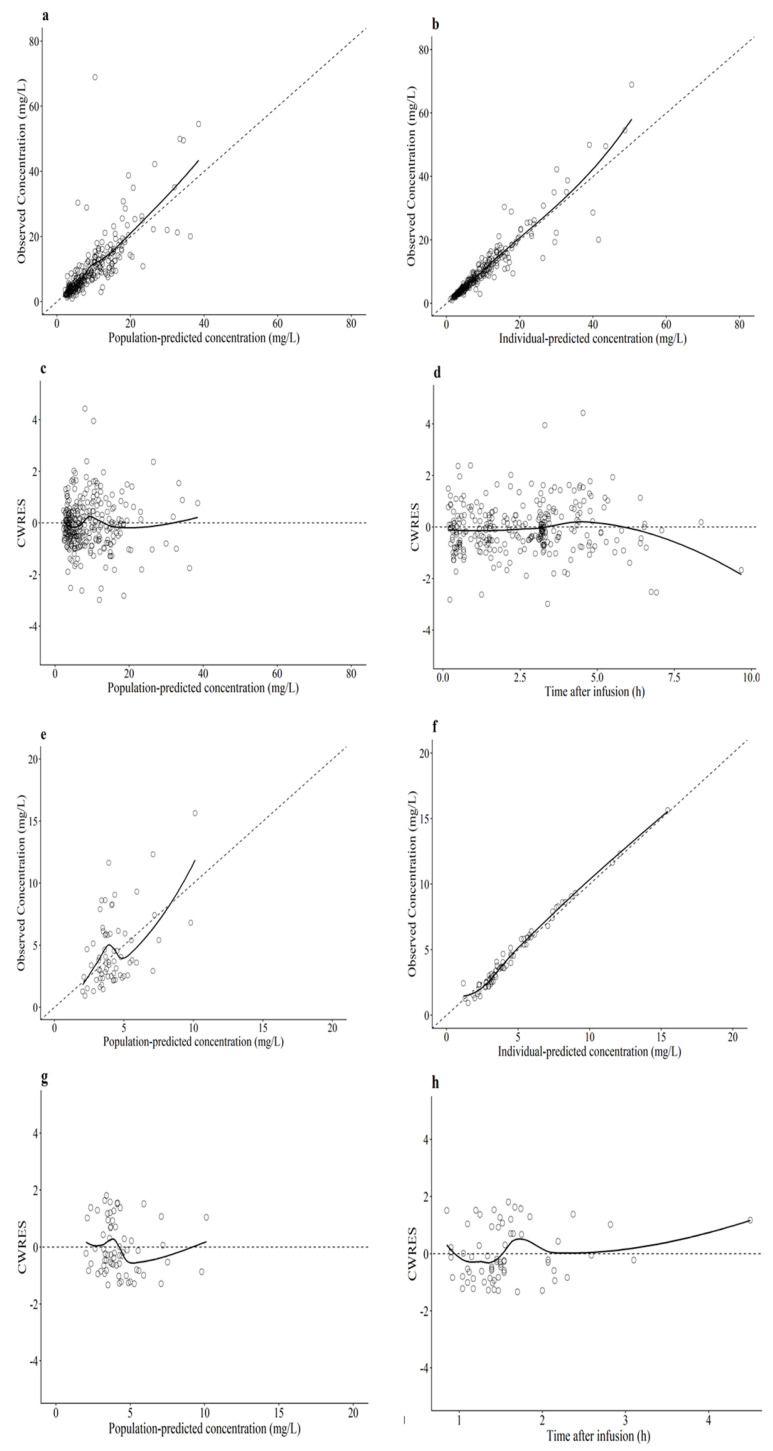
Goodness-of-fit plots for the final population pharmacokinetic model: (**a**,**e**) Observed versus population-predicted concentrations for unbound serum (**a**) and periprostatic adipose tissue (**e**). (**b**,**f**) Observed versus individual-predicted concentrations for unbound serum (**b**) and periprostatic adipose tissue (**f**). (**c**,**g**) Conditional weighted residuals (CWRES) versus population-predicted concentrations for unbound serum (**c**) and periprostatic adipose tissue (**g**). (**d**,**h**) CWRES versus time after infusion for unbound serum (**d**) and periprostatic adipose tissue (**h**). The solid lines represent the locally estimated scatterplot smoothing (LOESS) lines to assess model bias. The dashed lines represent the line of identity (y = x) for observed versus predicted plots (**a**,**b**,**e**,**f**) or the zero line for residual plots (**c**,**d**,**g**,**h**).

**Figure 3:**
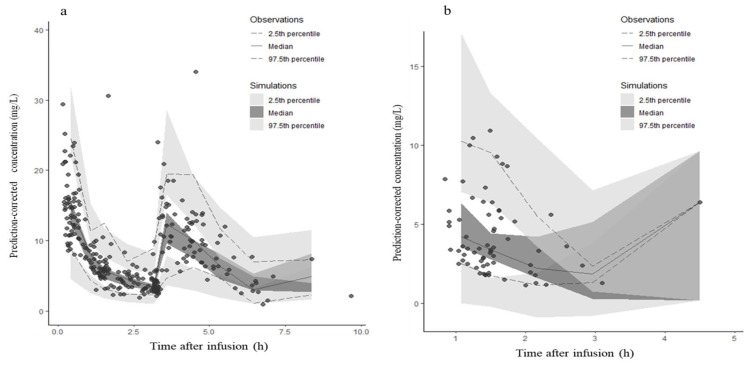
Prediction-corrected visual predictive check (pc-VPC) of the final model. Pharmacokinetic profiles of unbound serum (**a**) and periprostatic adipose tissue (**b**) cefazolin concentrations. Solid lines represent the observed median concentrations, and dashed lines represent the observed 2.5th and 97.5th percentiles. Shaded areas represent the 95% confidence intervals of the simulated 2.5th, 50th, and 97.5th percentiles.

The predictive performance of the final model was assessed using goodness-of-fit plots (Figure 2). Scatter plots of observed versus population-predicted concentrations (Figure 2a,e) and individual-predicted concentrations (Figure 2b,d) showed that the data points were distributed symmetrically around the line of identity. To evaluate potential model bias, conditional weighted residuals (CWRES) were plotted against population-predicted concentrations (Figure 2c,g) and time after infusion (Figure 2d,h). In these plots, locally estimated scatterplot smoothing (LOESS) lines (solid lines) were added to aid visual inspection. The LOESS lines were close to the zero line, and the residuals were symmetrically distributed across the concentration and time ranges, indicating no significant bias in the structural model or residual error model for both unbound serum and periprostatic adipose tissue concentrations. The prediction-corrected visual predictive check (pc-VPC) is presented in Figure 3a,b.

**Figure 4:**
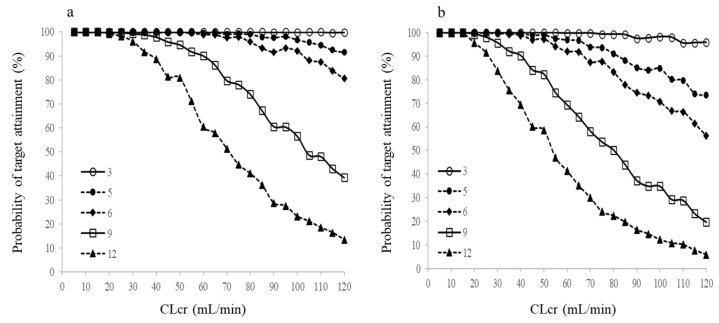
Monte Carlo simulations showing the probability of target attainment for the minimum inhibitory concentration (MIC). Trough concentrations in unbound serum (**a**) and periprostatic adipose tissue (**b**) following 1 g doses administered over 15 min, across creatinine clearance (CLcr) values of 5–120 mL/min at 3, 5, 6, 9, and 12 h post-administration.

In the bootstrap analysis of the final model, 975 out of 1000 bootstraps yielded successful results. The parameter estimates obtained from the bootstrap analysis were similar to those of the final model (Table 2). Subsequently, we performed a simulation using the final model to determine the optimal dosing regimen for patients with renal impairment. We simulated the unbound trough concentrations of CFZ for 1000 patients with CLcr values ranging from 5 to 120 mL/min. Figure 4 illustrates the probability of maintaining unbound trough concentrations of CFZ > 0.5 mg/L, a target associated with effective protection against MSSA. The probability of target attainment (PTA) values showed a decreasing trend with increasing CLcr and time after administration. Specifically, patients with higher CLcr exhibited a more rapid decline in PTA, reflecting the faster elimination of CFZ. Notably, the simulations indicated that for patients with mild renal impairment (CLcr ≤ 80 mL/min), a PTA of >90% was maintained for up to 5 h, suggesting that the standard redosing interval could be safely extended in this subgroup.

## 3. Discussion

While population pharmacokinetic modeling of CFZ has been conducted in various surgical settings, data on CFZ distribution into periprostatic adipose tissue—a critical site for infection prevention in urologic surgery—remain scant. This study represents the first pharmacokinetic characterization of CFZ in this specific tissue. By applying established modeling frameworks for beta-lactam tissue distribution, we evaluated the validity of the recommended dosing interval for patients undergoing RARP.

The unbound CFZ concentration profiles showed substantial interindividual variability, with a trend similar to that observed in previous studies involving perioperative patients [15]. No prior studies have measured CFZ concentration profiles in periprostatic adipose tissue; therefore, direct comparisons were not possible. Pevzner et al. reported a subcutaneous fat concentration of 9.37 µg/g during surgery in patients undergoing caesarean section who received 2 g CFZ, noting relatively low concentrations in patients with obesity [16]. Similarly, another study in patients with obesity reported a fat concentration of 6.66 µg/mL after a 2 g CFZ dose [17]. Considering that the average periprostatic fat concentration in our study was 4.54 µg/g and that >80% of participants received a 1 g dose, CFZ may show comparable or even higher tissue penetration in periprostatic fat than previously reported. Kinetic modeling strategies for tissue distribution often differ depending on the drug class. Unlike fluoroquinolones, which are substrates for active efflux transporters in prostatic tissue and thus require complex modeling incorporating Michaelis–Menten kinetics, as demonstrated by Hurtado et al. [18], the distribution of beta-lactams such as CFZ into adipose tissue is primarily driven by passive diffusion. Wittau et al. compared structural models for tissue distribution and demonstrated that a rapid equilibrium model adequately described the tissue pharmacokinetics of beta-lactams [19]. Therefore, the partition coefficient model used in this study is considered the most appropriate and robust approach for describing CFZ tissue penetration in the absence of active transport mechanisms.

High CFZ penetration into prostatic tissue has been reported, with prostatic tissue concentrations of 80.2 ± 38.6 µg/g compared with serum concentrations of 126.5 ± 19.1 µg/mL [20]. High prostatic tissue penetration has also been observed with cefazedone, a similar first-generation cephalosporin [21]. These findings support our hypothesis that prostatic tissue and its surrounding fat exhibit higher CFZ penetration than other adipose tissues.

The physiological plausibility of our model is supported by the identification of CLcr as a significant covariate for clearance, consistent with the primary renal elimination of CFZ [9]. This alignment with the findings of previous population pharmacokinetic analyses of unbound CFZ [22,23,24] reinforces the reliability of using CLcr as a guiding parameter for dosing adjustments. Our simulations confirmed the robustness of the current standard regimen for patients with normal renal function, demonstrating that a 3 h redosing interval consistently achieves a PTA of >90%. This finding aligns with the general pharmacokinetic principle that redosing at approximately twice the elimination half-life (typically 1.8–2 h for cefazolin) is effective for maintaining therapeutic levels [25]. The simulation results also highlighted an inverse relationship between CLcr and PTA. This trend underscores that while standard dosing is sufficient for patients with renal impairment, careful monitoring or shorter dosing intervals might be necessary for patients with augmented renal clearance to maintain effective concentrations.

Crucially, our analysis identified a distinct subgroup where the standard protocol may be overly conservative. Patients with CLcr ≤ 80 mL/min consistently maintained target concentrations for at least 5 h. As the operative time for RARP typically ranges between 3.4 and 4.8 h [26,27], this 5 h window of protection is clinically sufficient for this specific procedure. Therefore, while CLcr is a continuous covariate, the threshold of 80 mL/min serves as a practical decision point for RARP: our simulation results suggest that pharmacodynamically effective concentrations are maintained for up to 5 h in patients with CLcr ≤ 80 mL/min, and a universal additional dose may not be required. These findings provide a pharmacokinetic rationale for re-evaluating the necessity of the strict 3 h redosing protocol in this specific population.

This study had a few limitations. First, the limited number of patients weighing >80 kg (*n* = 8) suggests that the dosing regimen for this group may be insufficient. Second, data collected on periprostatic adipose tissue concentrations from only a single time point may lead to limited insights into their progression. Developing a complex kinetic model, such as the three-compartment model described by Hurtado et al. [18], was not feasible because of our sparse sampling design. However, given that a previous study has reported rapid tissue equilibration for similar antibiotics [19], we believe that the partition coefficient model adopted in this study provides a reasonable and practical estimation of tissue exposure despite the limited sampling points. Third, as this study primarily focused on patients undergoing RARP, who tend to be older, the predictive accuracy for younger patients or those with unimpaired renal function may be limited. Finally, caution should be exercised when applying the dosing intervals derived from this study to clinical practice. Our findings are based on pharmacokinetic simulations targeting surrogate endpoints (time > MIC), and we did not assess the actual SSI rates. Therefore, while our data suggest that extending the interval may be pharmacokinetically feasible, the clinical safety of this strategy regarding infection prevention remains to be confirmed in future prospective studies.

In conclusion, despite these limitations, the model developed in this study may be useful for informing CFZ dosing regimens in patients undergoing RARP by integrating both unbound serum and tissue pharmacokinetics. We found that for patients with normal renal function, the current 3 h dosing interval appears to be adequate. Conversely, for patients with CLcr ≤ 80 mL/min, our model predicts that sufficient concentrations would be maintained even with a dosing interval of 5 h. While these results indicate that the current guideline might be conservative for patients with mild renal impairment, future prospective studies are warranted to validate whether extending the redosing interval maintains low SSI rates in a clinical setting.

## 4. Materials and Methods

### 4.1. Study Design and Ethics

This prospective cohort study was conducted at Kitasato University Hospital, Kanagawa, Japan, a tertiary-care facility. Patients aged ≥18 years who underwent RARP between December 2021 and February 2025 were included. Patients with a known history of hypersensitivity to cephalosporin antibiotics were excluded. Following induction of anesthesia and within 60 min prior to surgical incision, all patients received intravenous prophylactic CFZ over a 10 min period, in accordance with the American Society of Health-System Pharmacists guidelines [28]. Patients weighing <80 kg received 1 g CFZ, whereas those weighing ≥80 kg received 2 g. Intraoperative redosing was performed every 3 h, with the dose determined by patient weight (1 g for <80 kg and 2 g for ≥80 kg), consistent with the initial administration. All participants provided written informed consent prior to the procedure. This study was conducted in accordance with the tenets of the Declaration of Helsinki and was approved by the Ethical Review Board of our hospital (approval number: B21-148).

Blood samples were collected at the time of the initial incision, during prostate removal, at the time of redosing (for surgeries lasting ≥3 h), and before skin closure. Periprostatic adipose tissue was collected only during prostate removal. Blood samples were stored on ice immediately after collection and were centrifuged at 1000× *g* for 10 min within 1 h after the conclusion of surgery. All samples were promptly centrifuged following collection, and the resulting serum was stored at −80 °C until further analysis. All samples were subjected to identical freeze–thaw conditions to ensure consistency.

### 4.2. Measurement of Unbound and Periprostatic Adipose Tissue CFZ Concentrations

Unbound and periprostatic adipose tissue CFZ concentrations were measured using high-performance liquid chromatography, as previously described [29].

Unbound CFZ concentrations were measured by centrifuging 400 μL of serum at 10,000× *g* for 15 min using a Nanosep 10K centrifugal device (Pall Corporation, Port Washington, NY, USA), following a previously reported method [30]. Periprostatic adipose tissue samples were pulverized in liquid nitrogen using a Cryopress (Microtec Nition, Chiba, Japan) to yield powder. The powdered samples were then homogenized in 300 µL of phosphate buffer. The homogenate was centrifuged, and the supernatant was collected. The supernatant (100 µL) was mixed with 150 µL of methanol (vol/vol) and centrifuged at 10,000× *g* for 10 min. The resulting sample solutions (50 µL) were then injected into a C18 column maintained at 10 °C. Samples were separated using the mobile phase (detailed below) at a flow rate of 1.0 mL/min, and the eluate was monitored at a wavelength of 254 nm with an ultraviolet absorption detector. The mobile phase comprised 85% 0.01 M sodium acetate (pH 5.2) and 15% acetonitrile (96%)–methanol (4%) solution. The lower limit of detection for CFZ was 0.5 mg/L. The calibration curve was linear over the concentration range of 0.5–100 mg/L, with a coefficient of determination (R^2^) of >0.99. Both inter-day and intra-day coefficients of variation were <5%.

### 4.3. Population Pharmacokinetic Analysis

Population pharmacokinetic modeling was performed using NONMEM v.7.6 software (ICON Development Solutions, Ellicott City, MD, USA). The first-order conditional estimation with interaction (FOCE-I) method was used for analysis. To determine the optimal structural model for unbound serum concentrations, one- and two-compartment models were systematically evaluated. Pharmacokinetic parameters estimated depended on the model structure: unbound CL and volume of distribution (V) for the one-compartment model and CL, V_c_, V_p_, and Q for the two-compartment model. Residual variability was evaluated using proportional, additive, and combined error models. The optimal model was selected based on the AIC, decreases in the objective function value (OFV), and visual inspection of goodness-of-fit plots. Inter-individual variability was evaluated for all pharmacokinetic parameters using an exponential error model. If the estimation of inter-individual variability for a specific parameter resulted in model instability or negligible variance, it was fixed to zero. To simultaneously analyze periprostatic adipose tissue concentrations, we linked the tissue data to the central compartment of the selected serum model. We referred to existing literature for linking unbound and tissue concentrations and performed the following analysis [19].

Given the sparse tissue sampling (one point per patient), estimating distinct rate constants for tissue transfer was not feasible owing to model over-parameterization. Therefore, we assumed a rapid rate of tissue penetration, implying that tissue concentrations were in equilibrium with the central compartment. Accordingly, tissue pharmacokinetics were modeled using a partition coefficient (F_PA_), relating tissue concentrations directly to unbound serum concentrations in the central compartment. The following covariates were evaluated for their potential influence on pharmacokinetic parameters: age; body weight; bilirubin, albumin, and serum creatinine levels; and CLcr. CLcr was estimated using the Cockcroft–Gault formula. Continuous covariates were incorporated into the model using a proportional relationship, defined as follows:

θ_i_ = θ_pop_ × (Cov_i_/median[Cov])^β^,

where θ_pop_ represents the typical population value for parameters for a patient with the median covariate value; Cov_i_ denotes the specific covariate value for individual i, while β is the estimated exponent reflecting the influence of the continuous covariate, as determined using the modeling software.

Categorical covariates were incorporated into the model using a multiplicative relationship, defined as follows:

θ_i_ = θ_pop_ × β^Covi^

where Cov_i_ is a binary indicator (0 or 1).

The significance of covariate influence on parameters was determined by evaluating the change in the objective function value (OFV) obtained from the NONMEM fitting routine. The statistically significant model among hierarchical models was selected using an alpha level of 0.05, corresponding to a decrease in OFV exceeding 3.84 (*p* < 0.05; χ^2^ distribution; one degree of freedom [df] for each additional parameter). The robustness of the full model was established by including all significant covariates. The final model was then refined through a stepwise backward elimination process, where each parameter in the full model with an OFV change of <6.63 (*p* < 0.01; 1 df) was successively removed.

Model adequacy was evaluated through several diagnostic plots: observed versus predicted CFZ concentrations, individual predicted concentrations after each Bayesian step versus observed concentrations, and weighted residual concentrations versus predicted concentrations. A pc-VPC was conducted to qualify the final model and parameter estimates. For the pc-VPC, 1000 simulated replicates of the original dataset were generated using the final model. Nonparametric bootstrap analysis was employed using the Perl-speak-NONMEM software to assess the reliability and stability of the estimated parameters [31]. The final model was refitted with 1000 additional bootstrapped replicates. The mean, standard deviation, relative standard error (%), and 95% confidence intervals were derived from the empirical bootstrap distribution and were compared with the estimates obtained from the original dataset. Based on a previous report [32], the specific gravity of the periprostatic adipose tissue was assumed to be 1.0 (1 kg = 1 L) for the pharmacokinetic analysis.

### 4.4. Pharmacodynamic Monte Carlo Simulations

Monte Carlo simulations were performed using NONMEM with the simulation dataset to generate unbound concentrations at 3, 5, 6, 9, and 12 h after a single dose of CFZ (1 g) administered via a 15 min intravenous infusion, which reflects standard clinical practice. Based on a previous report [33], the pharmacodynamic target was defined as maintaining both the unbound serum concentration and the periprostatic adipose tissue concentration above the MIC for at least 90% of the dosing interval.

The target pathogen was MSSA, a key causative pathogen for SSI in RARP [34]. The MIC value for MSSA was the MIC_90_ of 0.5 mg/L, obtained from Japanese SSI surveillance studies, because these values are highly applicable to clinical settings [35].

## 5. Conclusions

We successfully developed the first pharmacokinetic model for CFZ using both unbound serum and periprostatic adipose tissue concentrations in patients undergoing RARP. The simulation results demonstrated that the currently recommended 3 h redosing interval achieved PTA exceeding 90% in this patient population with normal renal function. Furthermore, for patients in this cohort with mild renal impairment (CLcr ≤ 80 mL/min), the PTA remained >90% for up to 5 h. These findings provide a pharmacokinetic rationale for re-evaluating dosing intervals in this specific demographic; however, future prospective clinical studies are required to validate the safety and efficacy of this strategy regarding infection prevention and to assess its applicability to broader surgical populations.

## Figures and Tables

**Table 1:** Patient characteristics.

	No.	Median (IQR)
Number of patients	67	
Age (years)		69 (63–73)
Weight (kg)		67.2 (60.4–74.2)
Serum creatinine (mg/dL)		0.92 (0.83–1.06)
Creatinine clearance (mL/min)		69 (60–88)
Albumin (g/L)		32 (30–34)
Total bilirubin (µmol/L)		17.1 (13.7–23.1)

IQR, interquartile range.

**Table 2:** Summary of the modeling information of the population included in the pharmacokinetic analysis.

Description	Parameter	Final Model		Bootstrap (*n* = 1000)		
		Estimate	RSE (%)	Median	Lower 2.5%	Upper 97.5%
Structural model parameters *						
CL (L/h) = θ1 × (CLcr/69)^θ2^	θ1	25.8	5.47	25.5	21.6	28.3
	θ2	0.735	19.46	0.738	0.459	1.066
V_c_ (L) = θ3	θ3	44.3	10.18	44.6	32.2	52.5
Q (L/h) = θ4	θ4	44.0	11.84	43.2	35.6	65.1
V_p_ (L) = θ5	θ5	52.2	8.85	53.1	44.2	68.9
F_PA_ = θ6	θ6	0.638	6.68	0.636	0.560	0.724
Between-subject variability						
ωCL (%)		28.3	34.00	29.0	19.3	39.1
ωV_c_ (%)		52.9	51.11	50.7	25.0	77.7
ωV_p_ (%)		46.3	53.74	46.0	10.9	70.6
FPA		45.0	16.04	44.4	37.3	51.1
Residual unidentified variability						
ε_pro_		21.6	8.79	21.6	18.1	25.3

* CL, unbound clearance; V_c_, unbound volume of distribution of the central compartment; Q, unbound intercompartmental clearance; V_p_, unbound volume of distribution in the peripheral compartment; ω, square root of inter-individual variance; F_PA_, factor for periprostatic adipose tissue; ε_pro_, residual variability for proportional error; RSE, relative standard error of the estimate.

## Data Availability

The data that support the findings of this study are available from the corresponding author upon reasonable request.

## Abbreviations

The following abbreviations are used in this manuscript:

CFZ	Cefazolin
CL	Unbound clearance
CLcr	Creatine clearance
CWRES	Conditional weighted residuals
df	Degrees of freedom
FOCE-I	First-order conditional estimation with interaction
F_PA_	Factor for periprostatic adipose tissues
MIC	Minimum inhibitory concentration
MSSA	Methicillin-susceptible *Staphylococcus aureus*
OFV	Objective function value
pc-VPC	Prediction-corrected visual predictive check
Q	Unbound intercompartmental clearance
RARP	Robotic-assisted radical prostatectomy
SSI	Surgical site infection
V_c_	Unbound volume of distribution in the central compartment
V_p_	Unbound volume of distribution in the peripheral compartment
